# How Effective Are the Nance Appliance and Transpalatal Arch at Reinforcing Anchorage in Extraction Cases?

**DOI:** 10.7759/cureus.61171

**Published:** 2024-05-27

**Authors:** Rawan Alrehaili, Ashraf Alhujaili, Waleed Almanjhi, Huda Alnami, Saleha Alsaiyari, Hanadi Alqahtani, Reem Alabdan, Dalia Baamer, Ahmed Khalil

**Affiliations:** 1 Dentistry, Private Practice, Medina, SAU; 2 Dentistry, Primary Health Care, Medina, SAU; 3 Dentistry, King Khalid University, Abha, SAU; 4 Dentistry, Ministry of Defense, Dhahran, SAU; 5 Dentistry, Armed Forces Hospital, Khamis Mushait, SAU; 6 Dentistry, King Abdulaziz University, Jeddah, SAU; 7 Orthodontics, Private Practice, Alexandria, EGY

**Keywords:** anchorage, orthodontic anchorage, nance, tpa, transpalatal arch

## Abstract

Objective: This narrative review aimed to evaluate, based on current evidence, whether the transpalatal arch (TPA) and Nance appliance can effectively reinforce anchorage during fixed orthodontic treatment while also offering a comprehensive and in-depth overview of the existing literature on this subject.

Materials and methods: A thorough literature search was performed across multiple electronic databases to identify peer-reviewed articles relevant to the review.

Results: Evidence suggests that the Nance appliance does not provide absolute anchorage. Additionally, patients experienced discomfort and inflammation of the palatal tissues. The transpalatal arch is also insufficient for maximum anteroposterior anchorage, and existing studies on its effectiveness in vertical anchorage control are inconsistent with conflicting data.

Conclusions: For patients with critical anchorage demand, mini-screws may be the method of choice, either solely or in combination with Nance or transpalatal arch, though they carry a risk of failure.

## Introduction and background

Orthodontic anchorage is defined as the resistance to unwanted tooth movement [1]. When a force is applied to move teeth in a particular direction, it results in an equal force being exerted in the opposite direction. This results in the potential for unwanted tooth movement, also referred to as anchorage loss [1]. Moyers [2] provided a clear definition of anchorage by dividing it into single, compound, and reinforced types. Subsequent researchers developed their own classification systems. Gianelly and Goldman [3] introduced the terms maximum, moderate, and minimum to describe the extent of movement expected in active and reactive tooth units under force. Marcotte [4] and Burstone [5] categorized anchorage into groups A, B, and C based on how much each unit contributes to space closure. Cope [6] emphasized the importance of anchorage preparation, including uprigtening or distal tipping of posterior teeth, to leverage the mechanical advantage before anterior retraction. Anchorage control is crucial in orthodontics as it enables precise movement of targeted teeth while maintaining the position of other teeth to serve as a stable base. Effective anchorage management ensures that the desired orthodontic goals, such as closing extraction spaces or aligning impacted teeth, are achieved without unwanted movements that could compromise the overall treatment outcome. Proffit et al. [1] highlighted that inadequate anchorage leads to undesirable shifts of anchoring teeth, resulting in complications like molar mesialization when trying to significantly retract anterior teeth. Moyers [2] emphasized that strong anchorage control reduces treatment time by minimizing unintended corrections, thereby improving patient comfort and satisfaction. Different methods to minimize undesired tooth movements have been proposed, including incorporating multiple teeth [7,8], the use of headgear [9-12], protraction face masks [13-15], transpalatal arch (TPA) [16-18], Nance buttons [19-21], lingual arch [22-24], elastics [25], or buccal or palatal skeletal anchorage [26-28].

The TPA was first introduced by Robert Goshgarian [29]. It is an appliance that consists of a stainless steel wire, usually 0.9 mm in diameter, that is soldered to the maxillary first molars by bands and crosses the hard palate. It is believed that it reinforces anchorage by preventing the mesial movement of molars. The incorporation of a U-loop in the midline directed anteriorly increases the flexibility of the wire for correct placement [30].

Different tooth movements are possible with a TPA, including derotation of unilateral or bilateral rotated molars [31-33], correction of molar crossbite [34], and intrusion of overerupted molars [35]. Additionally, TPA was utilized for asymmetric distalization of teeth and for applying buccal or lingual root torque to the upper molars [36]. The Nance palatal arch (NPA) is a modified version of TPA, composed of a stainless steel wire that incorporates an acrylic button. This acrylic button is strategically placed to enhance anchorage by resting against the cortical bone of the hard palate, thereby reinforcing the appliance's stability [37].

Loss of anchorage during orthodontic treatment can extend treatment time and undermine the overall effectiveness, which may result in poor treatment outcomes. TPA and NPA are two of the most frequently utilized devices for anchorage reinforcement, favored for their straightforward application and non-invasive nature. Given their widespread use, it is imperative to rigorously assess the effectiveness of the NPA and TPA in reinforcing anchorage to ensure they deliver the desired objective efficiently. This review aims to evaluate, based on the current evidence, whether TPA and NPA can be used for anchorage reinforcement during fixed orthodontic treatment and provide a thorough overview of the existing literature on this subject.

## Review

Fundamentals of anchorage in orthodontics

Tooth movement initially increases with rising force, but only up to a certain threshold, after which further force can strain the anchor units and negatively affect anchor teeth [1]. Optimum force is defined as the amount that elicits a maximum or near-maximum response, and its magnitude depends on the desired tooth movement [1]. Applying force beyond these levels can overcompress the periodontal ligament, causing cell death, hyalinization, and root resorption, ultimately compromising the desired outcome [2].

The characteristics of orthodontic appliances, including material, deflection, length, and thickness, are critical factors in determining the levels of force applied. A light, continuous, gentle force is most effective for achieving the intended movement, typically taking effect about 14 days after the compression of the periodontal ligament. This helps explain why fixed appliances are highly effective in comparison to alternatives like clear aligner therapy [3].

Proper anchorage is essential for achieving accurate tooth movement by concentrating force where needed and distributing reaction forces across multiple anchor teeth. The differential force theory posits that tooth movement is influenced by the tooth's surface area, with larger root surfaces providing greater resistance and, thus, stronger anchorage [4].

Simple anchorage involves pitting one tooth against another, while compound anchorage leverages multiple teeth in an anchor block, providing differential movement, particularly for less-supported sections [1-3]. This can be set up within a single arch or between both arches. Inter-maxillary anchorage, which utilizes elastics, springs, or functional appliances, balances forces across arches to prevent unwanted mesial movements and enhance occlusal stability, relying heavily on patient compliance [6].

Reciprocal anchorage distributes forces equally across teeth of similar sizes to close spaces like diastemas, effective only when the involved teeth have equal root surface areas [7]. Reinforced anchorage adds additional teeth to an anchor block to distribute reaction force more widely, typically used in scenarios like anterior tooth retraction into premolar spaces. Meanwhile, stationary anchorage employs techniques from the Begg method, facilitating the bodily movement of one anchor group against another by incorporating anchor bends in the wire [8].

**Figure 1:**
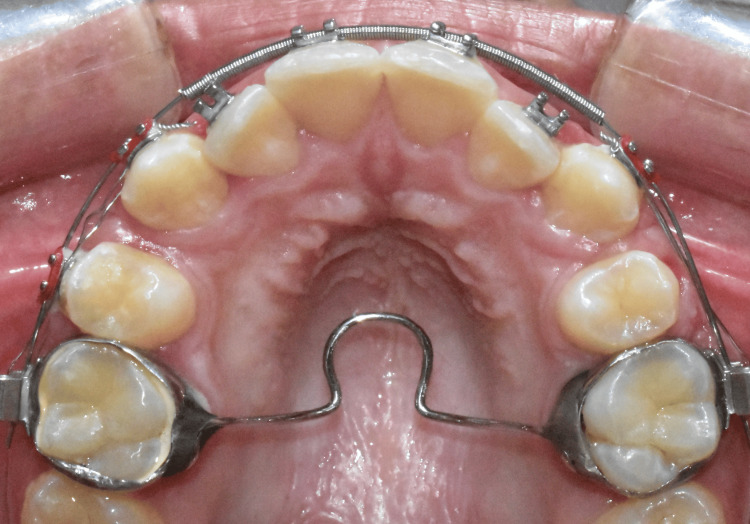
The use of a transpalatal arch with a U-loop in the midline in an upper first premolar extraction case Image credits: Ahmed Khalil

The TPA is crafted from a stainless steel wire, which is soldered to bands on the maxillary first molars and spans across the posterior hard palate (Figure 1).

**Figure 2:**
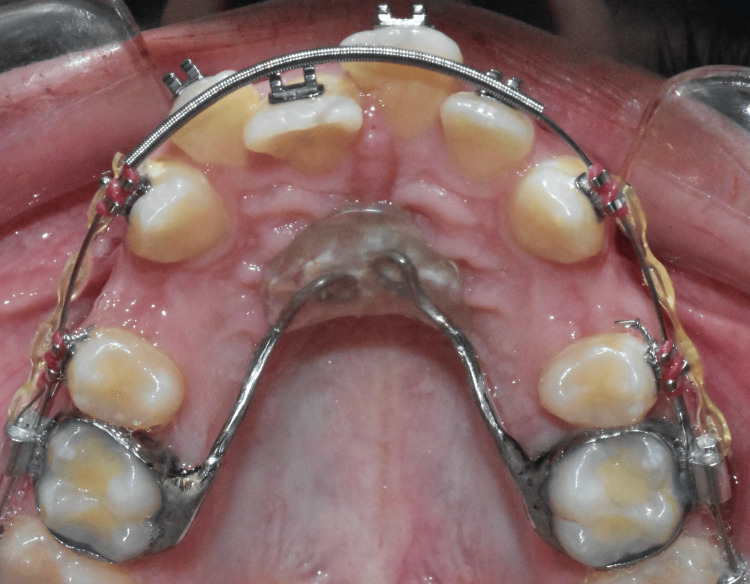
Implementing Nance appliance with an acrylic button resting against anterior palatal mucosa in an upper first premolar extraction case Image credits: Ahmed Khalil

In contrast, the NPA is a variation of the TPA, featuring a similar stainless steel wire but enhanced with an acrylic button for additional support using anterior palatal tissues (Figure 2).

Leveraging the Nance appliance for reinforcing anchorage

The pursuit of maximum anchorage is critical in managing tooth movement and achieving desired treatment outcomes in extraction as well as distalization cases. Recent studies have explored various methods of anchorage reinforcement during orthodontic treatment, demonstrating varied effectiveness across techniques. These methods are compared through different experimental designs, including split-mouth clinical trials, randomized clinical trials, multicenter research, and animal trials. A split-mouth clinical trial was conducted, including 14 subjects, to compare anchorage loss with NPA using both tipping and bodily mechanics [38]. The findings showed anchorage loss of 1.2 ± 0.3 mm and 1.4 ± 0.5 mm for tipping and bodily mechanics, respectively, indicating limited efficacy of NPA in providing absolute anchorage, accounting for 17% to 20% of total extraction space. In cases with high anchorage demands, the loss of a quarter of the extraction space could potentially compromise treatment outcomes. A viable solution to this issue could be the distalization of teeth, which, however, comes at the cost of increased treatment duration. Similarly, Arantes et al. [19] assessed the effectiveness of NPA and mini-implants (MI) in a randomized clinical trial with 18 patients. They found no significant difference between the methods, with mesial movements ranging from 1.90 mm to 2.85 mm, suggesting a comparable level of effectiveness in reinforcing anchorage during initial canine retraction. This indicates that both types of anchorage can be considered viable options in clinical practice, with the choice likely depending more on patient-specific factors and practitioner preference rather than any significant difference in clinical efficacy between the two methods. Complementing these findings, a multicenter randomized trial compared three anchorage reinforcement methods: NPA, MI, and headgear (HG) [20]. The trial included 87 subjects and reported average mesial movements from 0.80 mm to 2.09 mm. Although the differences were not statistically significant, MI showed superior treatment quality, hinting at its potential as the preferred method for achieving maximum anchorage. This finding stresses the superiority of MI in cases where critical anchorage preparation is required, particularly with severe crowding cases and sagittal discrepancies. Additionally, the primary challenge with using headgear in orthodontic treatment is ensuring patient compliance, which adds a significant burden to the patient. Further reinforcing these observations, a study conducted on beagle dogs aimed to evaluate NPA relative to a control group with no anchorage reinforcement and found significantly lower anchorage loss with NPA after 15 weeks [21]. Contrasting this, a clinical trial involving 50 patients noted that indirect anchorage using MI (group II) markedly outperformed NPA combined with a lingual arch (group I), with the latter groups experiencing anchorage losses up to 30%, while group II showed a minimal loss of 2.86% [39]. It is worth mentioning that the minimal anchorage loss reported with MI might be clinically insignificant. This phenomenon could be related to the inherent minor movements of the MI themselves.

Evidence highlighted a trend towards the efficacy of MI in anchorage reinforcement. This suggested that while NPA offers some reduction in anchorage loss, MI might provide a more reliable and effective solution in orthodontic cases requiring critical anchorage control. The challenge with NPA may arise from the Nance acrylic button resting on the firm anterior palatal tissues, which could contribute to patient’s discomfort. The design and placement of the acrylic button are critical, as improper fitting can lead to irritation or ulceration of the palatal tissues, influencing both comfort and the willingness of patients to adhere to treatment protocols.

Utilizing transpalatal arch for anchorage reinforcement

The efficacy of TPA in controlling anchorage during canine and anterior tooth retraction has been extensively studied, but the findings have been consistent to some extent. A comparative analysis study found that TPA did not effectively minimize anchorage loss in continuous arch mechanics during canine retraction, noting an anchorage loss of 4.5 mm [18]. Similar results were observed by Zablocki et al. [17] and Radkowski [16], who also reported that TPAs did not significantly improve molar anchorage in either the anteroposterior or vertical directions. In contrast, Kecik [40] found no significant difference in vertical anchorage control between MI and TPA. Yet, a significant anchorage loss anteroposteriorly, averaging 2.4 mm, was found when using TPA. Moreover, during the canine retraction period, no statistically significant differences were observed in the average mesial movement of molars between the two groups, one using palatal implants and the other combining TPA with a utility arch [41,42]. Vertical anchorage loss was noted in the form of molar extrusion during en-masse or two-stage retraction, and a decrease in inter-molar distance was reported after incisor retraction using TPA or even MI [43,44]. However, by the end of the anterior retraction phase, the palatal implants demonstrated superior anchorage control [45,46]. The limitations of TPA were further highlighted in the findings of Diar-Bakirly et al. [47], who conducted a systematic review and meta-analysis on the effectiveness of TPA as an anchorage method during anterior teeth retraction. They included 15 studies and found that using TPA alone resulted in significant mesial molar movement ranging from 27% to 54%. However, combining TPA with other anchorage methods reduced this movement from 20% to 40%. This indicates that TPA alone does not provide maximum anchorage, particularly during en-masse or two-step retraction, and the effectiveness of TPA combinations for en-masse anterior retraction remains uncertain, with evidence ranging from low to very low quality. Supporting these conclusions, Alharbi et al. [48] in their systematic review and meta-analysis compared TPA with conventional anchorage methods and MI, reporting a higher anchorage loss with TPA than with MI, averaging 3.13 mm. This suggests that MI might be a more effective method for reinforcing anchorage in orthodontic treatments requiring critical anchorage control.

In a randomized trial by Prado et al. [49], the stability of surgically assisted rapid palatal expansion was evaluated with and without the use of a transpalatal arch for retention. The study involved 30 adult patients divided into two groups: one with TPA as an anchorage and a control group. The findings suggested that retention with a TPA may not be necessary to maintain maxillary skeletal stability following SARPE. In a follow-up study comparing TPA versus control, the analysis indicated no superiority of using TPA for anchorage [50]. In the same vein, Raucci et al. [51] found a significant change in arch dimensions with the use of TPA. Conversely, a randomized clinical trial by Baccetti et al. [52] investigated the effects of rapid maxillary expansion (RME) and TPA combined with deciduous canine extraction (EC) on the eruption of palatally displaced canines (PDCs). The study involved 120 subjects, divided into three treatment groups and a control group. The results showed that the prevalence of successful canine eruptions was highest in the RME/TPA/EC group (80%) and the TPA/EC group (79.2%), compared to 62.5% in the EC group and 27.6% in the control group. This study concluded that TPA, even without RME, was effective in preventing canine impaction, providing a less invasive treatment option with similar success rates.

Combining TPA with MI has shown the potential to enhance treatment stability and effectiveness. In a prospective clinical trial, the combination of MI and TPA was utilized. The TPA functioned as an indirect stationary anchorage for the buccal segment [53]. In a related study, Felicita and Wahab [54] investigated the use of MI for the intrusion of maxillary posterior teeth in combination with TPA. The study demonstrated that significant intrusion could be achieved using a single buccal MI positioned bilaterally. The TPA was found to be successful in preventing buccal flaring of teeth. In the context of orthodontic anchorage, TPA has been explored for its efficacy and innovative applications. Shetty et al. [55] presented a modification of the TPA integrated with a fixed twin-block appliance to enhance retention and prevent accidental ingestion. This technique involved bending the free ends of the TPA occlusally into the acrylic bite blocks of the twin-block appliance for retention and anchorage.

The body of evidence collectively portrays a complex perspective on the effectiveness of TPA. It suggests that its utility might be constrained without supplementary anchorage reinforcement, especially in complex orthodontic scenarios that demand rigorous molar movement control and significant anterior tooth retraction.

Comparative analysis, Nance appliance vs. transpalatal arch: Which is better in anchorage control?

In exploring the efficacy of palatal appliances in controlling anchorage during orthodontic treatment, a randomized clinical trial compared the NPA and TPA [56]. Contrary to expectations, the study found negligible differences between the two arch types in preventing mesial drift and distal tipping, highlighting a similar performance in anchorage control. Although both arches prompted a slight disto-palatal rotation, with the TPA inducing a marginally higher degree of rotation control, these variations were minimal and likely of limited clinical importance. This suggests that the selection between the NPA and TPA should consider individual patient needs, supporting a personalized approach to orthodontic anchorage strategy.

Patient considerations

When using TPA and NPA for anchorage reinforcement, several patient considerations are crucial for successful orthodontic treatment. Comfort and adaptability to the appliance are significant, as both devices may cause initial discomfort due to their positioning close to the palatal tissues. The acrylic button of the NPA may sometimes lead to tissue irritation, inflammation, or even tissue necrosis in sensitive patients, requiring vigilant monitoring and potential removal if complications arise [27]. The TPA, on the other hand, is generally more comfortable, but maintaining proper hygiene is important to prevent plaque accumulation around the wire. Additionally, compliance with post-fitting instructions is essential to ensure that patients avoid any habits that could dislodge the device. Both appliances should be carefully monitored throughout treatment to ensure optimal performance in providing the desired anchorage reinforcement while minimizing the potential side effects [57].

Patient education and understanding of the appliance's purpose and the importance of maintaining oral hygiene are critical for treatment success. Regular checkups allow practitioners to identify and resolve any issues that may arise. Overall, both appliances require careful consideration of individual patient needs and preferences for successful use [47].

Future directions

The future of orthodontic anchorage is set to be revolutionized by advancements in technology, materials, and evidence-based strategies. A significant trend in this domain is the innovation of MIs. These devices offer versatile skeletal support while minimizing patient discomfort [20]. Additionally, MIs offer an alternative anchorage solution for lingual orthodontic patients, as traditional options like NPA and TPA are difficult to use in conjunction with lingual appliances [58,59]. They are becoming increasingly integrated with digital imaging and 3D printing, allowing clinicians to precisely plan and position MIs, resulting in more predictable outcomes [60]. Incorporating 3D technology, such as cone beam computed tomography, into digital guides for MI placement is another promising development [61]. These imaging techniques offer detailed anatomical information, which is then used to design custom surgical guides. These guides enable precise MI insertion at optimal angles and depths, reducing the risk of root or structural damage and lowering failure rates. Studies confirm that this increased accuracy minimizes the chances of MI failure [61]. Moreover, more personalized, technology-driven, and minimally invasive orthodontic solutions in digital planning tools help ensure that MI placement aligns with the overall treatment strategy and the desired tooth movement, reducing the risk of mid-treatment complications [62]. This combination leads to more efficient and predictable results.

Additionally, low-level laser therapy is emerging as a promising technique for enhancing MI osseointegration. This improves stability and reduces the risk of failure that is frequently encountered with its use [63]. Meanwhile, 3D-printed components facilitate the fabrication of personalized anchorage solutions tailored to each patient's oral anatomy [61,62].

Limitations

It is important to acknowledge the limitations of this review. Given the significant methodological heterogeneity among the studies, the results should be interpreted cautiously. Moreover, blinding the clinician and the patient to the anchorage reinforcement method is hardly achievable. Additionally, the review only included studies published in English, potentially overlooking relevant research in other languages.

## Conclusions

Recent evidence indicates that the Nance appliance does not achieve absolute anchorage. The reported amount of anchorage loss varies, and the data available show conflicting results. Patients have reported slight discomfort, inflammation, or even tissue necrosis with this appliance, emphasizing the need for further well-conducted randomized clinical trials.

The transpalatal arch alone is insufficient when a maximum anteroposterior anchorage is required, whether for en-masse or two-stage retraction. Scientific evidence on the effectiveness of the TPA in vertical anchorage control is limited, and the few existing studies provide inconsistent results. For patients with critical anchorage demand, mini-screws may be the method of choice, either solely or in combination with Nance or transpalatal arch, though they carry a risk of failure.
